# Preceding Benign Paroxysmal Positional Vertigo as a Trigger for Persistent Postural–Perceptual Dizziness: Which Clinical Predictors?

**DOI:** 10.3390/audiolres13060082

**Published:** 2023-12-01

**Authors:** Augusto Pietro Casani, Nicola Ducci, Francesco Lazzerini, Nicola Vernassa, Luca Bruschini

**Affiliations:** Department of Surgical and Medical Pathology, ENT Section, Pisa University Hospital, 56122 Pisa, Italy; n.ducci@yahoo.it (N.D.); francilazzerini@gmail.com (F.L.); n.vernassa@ao-pisa.toscana.it (N.V.); l.bruschini@gmail.com (L.B.)

**Keywords:** persistent postural–perceptual dizziness, vertigo, benign paroxysmal positional vertigo, precipitant conditions, older patients, vestibular migraine, recurrences, risk factors

## Abstract

Objective: Persistent postural–perceptual dizziness (PPPD) is a syndrome described as secondary, when it is the consequence of an organic disorder (s-PPPD), or primary, when no somatic triggers can be identified. We evaluated a group of patients diagnosed as s-PPPD, with Benign Positional Paroxysmal Vertigo (BPPV) as the main somatic trigger, with the aim of identifying the predictive clinical elements of evolution towards PPPD. Study Design: Retrospective case review. Setting: Tertiary referral center. Patients: We evaluated 126 patients diagnosed with PPPD; 54 patients were classified as p-PPPD (43%) and 72 as s-PPPD (57%). Of these, 51 patients had BPPV as a somatic trigger of PPPD, and in this group, we evaluated the prevalence of some clinical features (age, sex, latency between the onset of BPPV and the final diagnosis, recurrence of BPPV and the presence of migraine headache) for comparison with a group of patients who suffered from BPPV without an evolution towards PPPD (control group). Results: In the group with PPPD secondary to BPPV, we found a significantly higher mean age and a longer latency between the onset of BPPV and the final diagnosis compared to the control group. No difference between the two groups was found regarding sex, recurrence rate and the presence of migraine headache. Conclusions: The parameters most involved as potential precipitants of PPPD after BPPV were the age of the patients and a long latency between the onset of BPPV and the final diagnosis; the mean age of the subjects who developed PPPD following BPPV was significantly higher. These findings lead us to emphasize the importance of the early identification and treatment of BPPV, especially in older patients.

## 1. Introduction

Persistent postural–perceptual dizziness (PPPD) was defined in 2017 [1] as a syndrome that unifies the clinical characteristics of chronic subjective dizziness, phobic postural vertigo, and related disorders. The latest version of the International Statistical Classification of Diseases and Related Health Diseases (ICD-11) [2] describes it as follows: persistent non-vertiginous dizziness, unsteadiness, or both, lasting three months or more. Symptoms are present most days, often worsening throughout the day, but they may wax and wane. Momentary flare-ups may occur spontaneously or with sudden movement. Affected individuals feel worst when upright, when exposed to moving or complex visual stimuli, and during active or passive head motion. These situations may not be equally provocative. Typically, the disorder follows occurrences of acute or episodic vestibular- or balance-related problems, but may follow non-vestibular insults as well [1,3,4]. Symptoms may begin intermittently and then consolidate [4]. PPPD is considered a chronic functional disease of the brain and a condition in which there is a prolonged and over-adaptive response to acute dizziness, although anxiety itself and other traumatic life events can be a trigger [1,4,5]; however, PPPD is not a structural or psychiatric condition [4]. Alterations in the functioning of the neural structures involved in managing postural control, locomotion and spatial orientation represent PPPD’s primary pathophysiological process [1,4]. The association between dizziness and psychiatric comorbidity, especially anxiety and depression, is well known [5]. PPPD may co-exist with structural and/or psychological disorders, but it is defined as a different entity. Patients with PPPD have a high burden of dizziness, associated with negative consequences on daily life [6]. 

Recently, PPPD was described as secondary, when it is the consequence of an organic disorder (s-PPPD), or primary, when somatic triggers cannot be identified (p-PPPD) [7]. Among the somatic triggers for s-PPPD, acute unilateral vestibulopathy (AUPV) and Benign Paroxysmal Positional Vertigo (BPPV), together with vestibular migraine (VM), are the most common [7,8,9,10]. AUPV manifests as an acute onset of sustained spinning vertigo associated with neurovegetative symptoms, lasting for at least 24 h, with no evidence of neurological or audiological symptoms [11]. Up to 50% of patients develop either continuous or paroxysmal dizziness after AUVP; this can result in chronic dizziness, disequilibrium, spatial disorientation and limitations in daily activities [12,13]. BPPV is now considered the most common peripheral vestibular disorder and is believed to be the leading cause of vertigo worldwide [14], with a lifetime prevalence of 2.4% and an incidence that increases with age [15,16,17]. BPPV is characterized by positional vertigo and nystagmus, provoked by changes in the position of the head with respect to gravity. The diagnosis of BPPV is usually not troublesome, and the outcome of treatment is very satisfactory with noninvasive methods such as Canalith Repositioning Maneuvers (CRMs) [14,16,17]. In the present study, we evaluated a group of patients diagnosed as s-PPPD—with BPPV as the main somatic trigger—and compared them with a group of patients affected with BPPV without evolution to PPPD, with the aim of identifying the predictive clinical elements of evolution towards PPPD. 

## 2. Materials and Methods

In the Neurotological unit of the ENT department (tertiary referral center, Pisa University Hospital, Pisa, Italy), we retrospectively evaluated the clinical records of a consecutive series of 126 patients diagnosed with PPPD, according to the Bárány criteria (Table 1) [1], during the period of October 2018 to October 2021. All these patients, after a general ENT examination (during which a Romberg and a Fukuda stetting test are performed) received an accurate history-taking (with the aim of identifying the previous presence of vestibular comorbidities), and they underwent a complete neuro-ontological examination, including the search for spontaneous and positional nystagmus (using infrared goggles), caloric testing, a video head-impulse test and cervical and ocular vestibular evoked myogenic potentials (c-VEMPs and o-VEMPs). All the patients underwent a hearing test (pure-tone audiometry and tympanometry). Caloric tests were performed according to a modified Fitzgerald–Hallpike technique; the external auditory canal was separately irrigated with 125 mL of warm (44 °C) and cold (30 °C) water in a 30 s period (7 min between each test) and responses were recorded through an infrared eye-tracking system (GN Otometrics, Taastrup, Denmark). Canal paresis (CP) was considered significant if >25%. Bilateral vestibular areflexia/hyporeflexia was diagnosed when a bilaterally reduced caloric response (sum of the bilateral maximal peak slow phase velocity on each side <6°/s) was recorded. The vHIT was performed using a dedicated device (ICS Impulse System; version 2.0, GN Otometrics, Taastrup, Denmark). The patient was asked to stare at an Earth-fixed target (a 3 cm-diameter spot located 1.5 m in front). Twenty low-amplitude (10°–20°) and high-velocity head impulses (50–250°/s) were then randomly administered to each side for every semicircular canal. The device software automatically calculated the average high-velocity VOR gain. The software also calculated the asymmetry index (AI%, normal values within 15%; this value resulted from our own data collected in a group of normal subjects age ranging from 20 to 80-years-old) between the right and left sides. AI was calculated as $AI=\left[\frac{\left(G_{L}-G_{R}\right)}{\left(G_{L}+G_{R}\right)}\right]\times 100$, where G_R_ denotes the right-sided mean gain and G_L_ denotes the left-sided mean gain. AC cVEMPs and bone-conducted (BC) oVEMPs were delivered at the midline forehead (Fz point), respectively, through a dedicated device (ICS Chartr EP 200, GN Otometrics, Taastrup, Denmark). 

We compared the clinical characteristics of 51 patients with s-PPPD with BPPV as trigger BPPV (group 1), with a consecutive series of 107 subjects diagnosed with BPPV in the same period but with no evolution to PPPD. Posterior Semicircular Canal-BPPV was diagnosed according to the following criteria [18]: (a) Recurrent attacks of positional vertigo or positional dizziness provoked by lying down or turning over in the supine position; (b) Duration of attacks <1 min; (c) Positional nystagmus elicited after a latency of one or a few seconds by the Dix–Hallpike maneuver or the side-lying maneuver (Semont diagnostic maneuver). The nystagmus is a combination of torsional nystagmus with the upper pole of the eyes beating toward the lower ear combined with vertical nystagmus beating upward (toward the forehead), typically lasting <1 min and (d) Not attributable to another disorder. Lateral Semicircular canal BPPV was diagnosed according to the following criteria [18]: (a) Recurrent attacks of positional vertigo or positional dizziness provoked by lying down or turning over in the supine position; (b) Duration of attacks <1 min; (c) Positional nystagmus elicited after a brief latency or no latency by the supine roll test, beating horizontally toward the undermost ear with the head turned to either side (geotropic direction changing nystagmus) and lasting <1 min. (d) Not attributable to another disorder. All the patients suffering from BPPV were successfully treated after 1–3 cycles of CRMs (Semont or Epley maneuver for BPPV of the posterior semicircular canal and the Gufoni maneuver for BPPV of the horizontal semicircular canal). 

We evaluated the following parameters: age, sex, latency between the onset of BPPV and the final diagnosis, the recurrence of BPPV and the presence of migraine headache (MH). For this latter parameter, we evaluated the presence of symptoms that fulfilled or did not fulfill the criteria for migraine headache (MH), according to the International Headache Society [19]. The other diagnoses eventually encountered in patients with s-PPPD were made according to the following criteria: AUVP was diagnosed as an acute vestibular syndrome characterized by the acute onset of sustained spinning or non-spinning vertigo associated with neurovegetative symptoms (namely nausea and vomiting), with symptoms lasting for at least 24 h without neurologic or audiologic symptoms—characterized by a spontaneous unidirectional horizontal-torsional direction fixed nystagmus enhanced by the removal of visual fixation, unilateral vestibular areflexia/hyporeflexia on a bithermal caloric test and positive head impulse test results in the direction opposite to the fast phase of the nystagmus. VM was diagnosed in accordance with the consensus document of the Classification Committee of the Bárány Society [20]. In case of suspected central vestibular involvement, a brain MRI was performed. Vertebro–Basilar TIAs was diagnosed in cases of isolated transient vertigo in patients with multiple vascular risk factors, severe trunk ataxia and signs of central vestibular involvement (gaze evoked or vertical nystagmus, impaired saccades) [21]. Ethical review and approval by the local Institutional Board (Comitato Etico Azienda Ospedaliero, Universitaria Pisana, Pisa, Italy) were waived for this study. Due to its retrospective nature, it was not set up as part of a research project. Furthermore, the study did not include new experimental diagnostic protocols, and the patients included in the study were diagnosed according to national guidelines. Written informed consent was obtained from all participants, and the study was conducted in accordance with the 1964 Declaration of Helsinki. 

### Statistics

The Shapiro–Wilk test was employed to assess the normality of the data distribution, and the significance level was set at *p* < 0.05. The distribution of qualitative data across the groups was analyzed using the Chi-square test and Fisher’s exact test, both of which are appropriate for analyzing non-normally distributed data. Additionally, the statistical analysis used to assess the correlation between dichotomous variables involved employing the Chi-Square test or the Fisher’s exact test, where appropriate.

To determine the mean differences between quantitative data, the nonparametric Wilcoxon signed rank test was employed, since the data did not follow a normal distribution. All statistical calculations were performed using SPSS (IBM SPSS Statistics 23.0, IBM, New York, NY, USA). A *p*-value of ≤0.05 was considered statistically significant.

## 3. Results

Of the 126 patients diagnosed with PPPD, 54 patients were classified with p-PPPD (43%) and 72 with s-PPPD (57%). In the latter, the vestibular triggers for s-PPPD were represented by BPPV (51 cases, 70.83%), in 39 affecting the posterior semicircular canal and in 12 the horizontal semicircular canal; VM (twelve cases, 16.6%); AUVP (four cases, 5.5%) and central vestibular disease (five cases, 6.9%). Three of the five patients suffering from central vestibular disease were affected by vertebro-basilar TIAs; the remaining two patients were affected by cerebral small vessel disease. 

**Figure 1:**
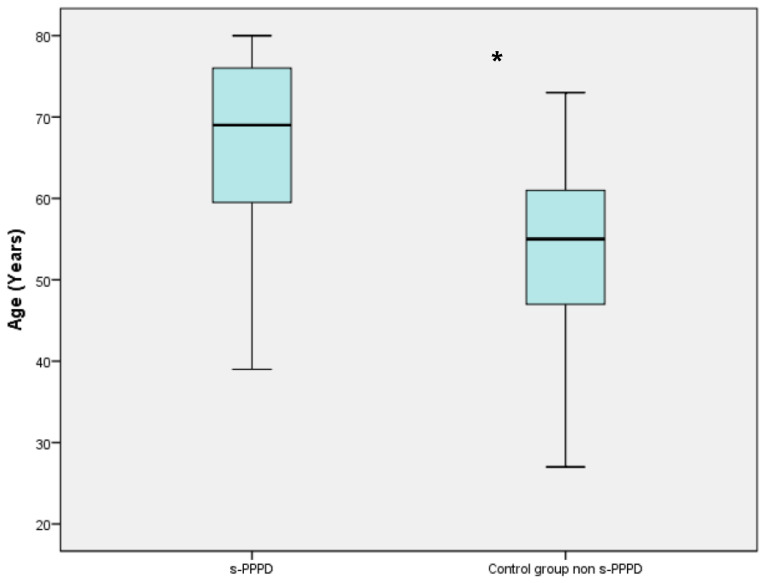
Boxplot chart showing the difference in mean age between the secondary persistent postural–perceptual dizziness (s-PPPD) group and the control group. The mean age of the s-PPPD group was 65.88 years (from 39 to 80 years); meanwhile, the mean age of the control group was 53.50 years (from 27 to 73 years). The box in the chart represents the interquartile range of the data; the line inside the box represents the median; the whiskers indicate the minimum and maximum values of the data. The difference is statistically significant with the Wilcoxon signed rank test (*; *p* < 0.001).

Of the 51 patients with s-PPPD post-BPPV, 34 (66%) were females and 17 (33%) were males, with a mean age of 65.88 years (39 to 80). The control group (patients with previous BPPV and no evolution to PPPD) consisted of 70 females and 37 males (mean age 53.50 ranging from 27 to 73). Table 2 shows the demographic data of the two studied groups (Table 1). The mean age of patients belonging to the s-PPPD group was statistically significantly higher compared to the non-s-PPPD subject group (*p* < 0.001), while no difference was found in the distribution of the sexes between the two groups (Figure 1). 

**Figure 2:**
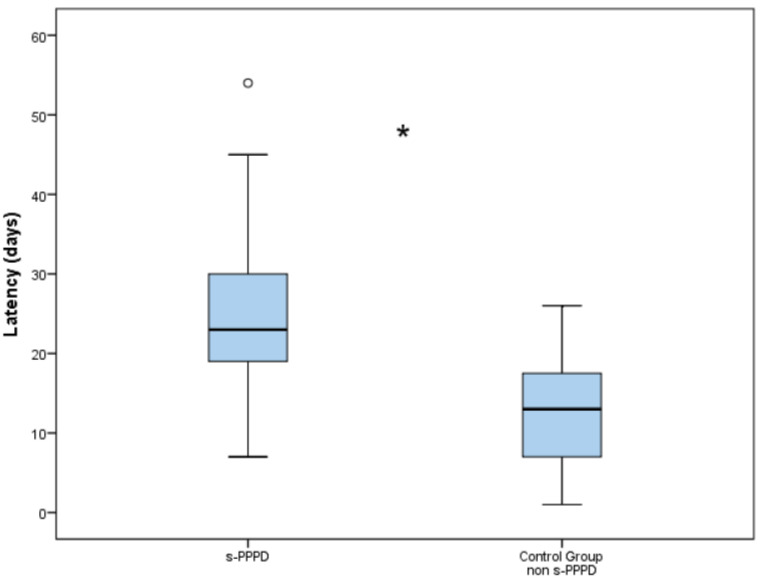
Boxplot chart showing the difference in latency (in days) between the secondary persistent postural–perceptual dizziness (s-PPPD) group and the control group. The mean latency in the s-PPPD group was 25.25 days (from 7 to 54 days); meanwhile, the latency in the control group was 12.77 days (from 3 to 26 days). The box of the chart represents the interquartile range of the data; the line inside the box represents the median; the whiskers indicate the minimum and maximum values of the data; outliers beyond this range are shown as individual data points (°). The difference is statistically significant with the Wilcoxon signed rank test (*; *p* < 0.001).

The latency between the onset of BPPV and the final diagnosis was 25.2 days (7 to 54) in Group 1 and 12.8 days (3 to 26) in the control group. The higher latency encountered in s-PPPD was statistically significantly higher compared to the non-s-PPPD group (*p* < 0.001; Figure 2). 

In total, 17 subjects (33%) in Group 1 and 37 (34.6%) patients belonging to the control group showed at least one episode of recurrent BPPV (evaluated over a period at least of 14 months). Finally, 8 (15.7%) patients in Group 1 and 14 (13.1%) in the control group showed a concurrent MH. No statistically significant differences were found between the s-PPPD group and the control group in the incidence of relapsing BPPV (*p* = 0.877) or MH (*p* = 0.659). No significant distribution differences were found between the sexes and the occurrence of relapsing BPPV (*p* = 0.607), MH (*p* = 0.801), or belonging to the s-PPPD group (*p* = 0.877). 

## 4. Discussion

In clinical practice, knowledge of PPPD predictors in patients suffering from BPPV and other vestibular pathologies could be very useful. Alongside the specific treatment for this pathological condition (namely, CRM), when predictive elements for PPPD occur, it will be extremely useful to combine specific treatments for PPPD, such as vestibular rehabilitation, medication and psychotherapy [3]. In fact, it has been demonstrated that early therapeutic intervention in PPPD is more effective [22]; this is particularly true in patients who present with comorbid anxiety disorders, a condition that naturally contributes to an evolution towards PPPD [23]. In the present study, we examined the clinical characteristics of patients who developed a secondary PPPD following a preceding episode(s) of BPPV as a potential somatic trigger. PPPD can arise without any evident somatic trigger or precipitating conditions, especially in the presence of psychiatric comorbidities; however, in about half of cases, PPPD is typically preceded by a disorder of the vestibular system, causing acute prolonged or episodic vertigo [9,10,14,15,16,17,18,19,20,21,22,23,24,25,26]. On the other hand, a high percentage of patients suffering from vestibular illness (including, among others, BPPV and VM) show a significant correlation with depression and anxiety disorders [27], which contribute to the worsening of their symptomatology—potentially leading to diagnostic errors and incorrect treatment.

In our series, the most common vestibular precipitant of PPPD was BPPV, and this result is in accordance with previously reported results [7,8,9,22]. On the contrary, the number of patients suffering from s-PPPD with previous VM was surprisingly low with respect to other experiences [9,28,29]; this result, as well as the finding of a high incidence of cases of PPPD secondary to BPPV, could be attributable firstly to the high prevalence of this disorder in the general population [15,30] and secondly to the peculiarity of our center, which could have a lower influx of patients with suspected neurological diseases such as VM. This consideration may also explain the finding of a significantly lower mean age in p-PPPD compared to the group of patients classified as s-PPPD. Furthermore, PPPD has been considered as a pre-existing spectrum in the nonclinical population, where high levels of PPPD symptoms have been found; vestibular damage could simply play the role of inducing the emergence of a pre-existing tendency to abnormal visuo-vestibular processing, leading to visually induced dizziness [22,31]. 

Taking into account that BPPV is the most common cause of dizziness/vertigo not only in the context of otoneurological clinical practice, but also in general practice and in the emergency department, it seemed extremely important to first correctly diagnose and treat this vestibular disorder, and secondly to evaluate whether there are some clinical elements that could induce the subsequent onset of PPPD. However, we need to separate s-PPPD subsequent to BPPV from so-called residual dizziness (RD). This condition could occur in up to two-thirds of patients despite successful treatment with a repositioning maneuver, and it may manifest as a prolonged and handicapping instability, lightheadedness and malaise [32,33]. The main difference between PPPD and RD is that the latter has a duration that generally does not exceed 20 days [32,33,34,35]—unlike PPPD, the criteria for which indicate a duration of more than three months [1]. In our experience, the parameters most involved as potential precipitants of PPPD subsequent to previous BPPV were represented by the age of the patients and a long latency between the onset of the positional vertigo and the final diagnosis; the mean age of the subjects who developed PPPD was significantly higher than the patients without evolution towards PPPD. A population-based study found that the one-year prevalence of BPPV in people older than 70 was nearly seven times that found in people younger than 40 [15]; geriatric patients with BPPV are more likely to have risk factors associated with atherosclerosis (such as hypertension, diabetes and hyperlipidemia, which may affect the blood supply to the inner ear) and osteopenia/osteoporosis [36]. In older adults, BPPV shows peculiar aspects and tends to have a less obvious or less characteristic presentation, as well as a more protracted course [37]—the latter probably due to a higher rate of cupulolithiasis, in which case, a lower positive response to CRM is observed [38]. BPPV in older adults tends to cause unsteadiness rather than a spinning sensation [39], which might be misleading regarding its differential diagnosis. All these conditions lead to a greater difficulty in achieving effective postural control, with the onset of a fear of falling, which can induce psychopathological reactions of anxiety and depression [16,40,41]. These considerations highlight the importance of the results of our study; the early diagnosis and treatment of BPPV is essential, especially in older adults, in order to avoid progression toward BPPV. 

The other main result of our study seems to demonstrate that diagnostic delay is the most strongly predictive element of an evolution towards PPPD. The finding in the s-PPPD patients of a significantly longer period between the onset of positional vertigo symptoms and the final diagnosis leads us to emphasize the importance of the early identification and treatment of this pathology, reassuring the patient about the benign nature of the disorder. This approach can significantly reduce the possibility that the patient, especially if affected by phobic–anxious personality traits, may develop a condition of chronic dizziness that can be classified as PPPD. A high percentage of patients do not perceive BPPV as a benign disease, causing a serious impact on their health-related quality of life and on their mental state [42]; the physical limitations caused by the disease and the anxiety and phobic avoidance of the precipitating head position could provoke an exaggerated emotional reaction, sometimes persisting for a long period after the vestibular symptoms have resolved [10,43]—especially in older adults [42,43]—inducing worse compliance with CRM. Our data confirm that the early detection and treatment of BPPV, together with reassurance about the benign nature of the disease, can undoubtedly decrease the risk of evolution to PPPD, as well as a reduction in the negative effects on various aspects of the patient’s social and occupational life (work, travel and social and family life) [44]. Regarding parameters such as the recurrence of BPPV and the presence of concomitant migraine headache (with no symptoms that could lead to a diagnosis of VM, as stated in the Section 2 of the manuscript), although the evidence of an epidemiologic relationship suggests an association between MH and BPPV and its role as a risk factor for the recurrence of BPPV [45], our data seem not to support the presence of MH as a predictor of developing PPPD. This latter observation seems to be in line with the results of a recent study in which it was demonstrated that the presence of VM in the BPPV patient’s history, but not migraine without VM, appears to increase the risk of developing PPPD in patients with BPPV [29]. Regarding the recurrence of BPPV, our results confirm the observations of Gambacorta et al. [10] affirming that PPPD occurs mainly in subjects who have suffered from an initial episode of BPPV. 

This study had several limitations: In our cohort, we did not assess the presence of anxious or depressive personality traits as possible risk factors for developing PPPD after BPPV. Other risk factors such as vascular risk factors, the presence or absence of tinnitus and/or hearing loss were not investigated in our study and should probably be considered when planning any future prospective study. It is well known that a high level of anxiety and an exaggerated vigilance towards acute dizzy symptoms are relevant conditions that induce the development of functional dizziness [3,4,5]. Therefore, the lack of an assessment of the patient’s psychological status at the time of diagnosis of BPPV is certainly a relevant bias. Another study limitation is represented by the characteristics of the study design; a prospective study with a large cohort of patients will surely introduce a better assessment of the parameters involved as risk factors favoring an evolution towards PPPD after a diagnosis of BPPV. 

## 5. Conclusions

PPPD is a condition characterized by chronic symptoms including subjective dizziness and instability that are typically exacerbated during upright self-motion and during exposure to complex full-field visual stimuli. BPPV is a common vestibular precipitant of PPPD that, in these cases, could be classified as secondary PPPD. Some parameters seem to be related to the evolution of BPPV into PPPD: the age of the patients and the latency between the onset of positional attacks of vertigo and diagnosis. For these reasons, the early identification and treatment of BPPV is a crucial clinical task in reducing the risk of evolution towards PPPD—especially in older patients—and it will be extremely useful to combine specific treatments for PPPD early-on such as vestibular rehabilitation, medication and psychotherapy.

## Figures and Tables

**Table 1:** Current diagnostic criteria of the Bárány Society for persistent postural–perceptual dizziness (PPPD).

(a) One or more symptoms of dizziness, unsteadiness, or non-spinning vertigo on most days for at least 3 months. Symptoms last for prolonged (hours long) periods of time, but may wax and wane in severity. Symptoms need not be present continuously throughout the entire day.
(b) Persistent symptoms occur without specific provocation but are exacerbated by three factors: Upright posture, active or passive motion—without regard to direction or position—and exposure to moving visual stimuli or complex visual patterns
(c) The disorder is precipitated by conditions that cause vertigo, unsteadiness, dizziness or problems with balance including acute, episodic or chronic vestibular syndromes, other neurologic or medical illnesses or psychological distress. When the precipitant is an acute or episodic condition, symptoms settle into the pattern of criterion as the precipitant resolves, but they may occur intermittently at first, and then consolidate into a persistent course. When the precipitant is a chronic syndrome, symptoms may develop slowly at first and worsen gradually.
(d) Symptoms cause significant distress or functional impairment.
(e) Symptoms are not better accounted for by another disease or disorder.

All five criteria (a–e) must be fulfilled to make the diagnosis of PPPD.

**Table 2:** Demographics and patient characteristics for the group of patients with PPPD secondary to BPPV and the control group (patients suffering from BPPV without evolution into PPPD). PPPD: Persistent Postural–Perceptual Dizziness. BPPV: Benign Positional Paroxysmal Vertigo.

	s-PPPD Group (51 Patients)	Control Group (107 Patients)	*p*-Value
Age, mean (range) years	65.88 (39–80) years	53.50 (27–73) years	<0.001
Sex	34 females (66%) 17 males (33%)	70 females (65.4%) 37 males (34.6%)	0.877
Latency between the onset of BPPV and the diagnosis, mean (range)	25.25 (7–54) days	12.77 (3–26)	<0.001
Recurrent BPPV	34 no (66.7%) 17 yes (33.3%)	70 no (65.4%) 37 yes (34.6%)	0.513
Migraine Headache	43 no (84.3%) 8 yes (15.7%)	93 no (86.9%) 14 yes (13.1%)	0.415

## Data Availability

Data available on request.

## References

[1] Staab J.P., Eckhardt-Henn A., Horii A., Jacob R., Strupp M., Brandt T., Bronstein A. (2017). Diagnostic criteria for persistent postural-perceptual dizziness (PPPD): Consensus document of the committee for the Classification of Vestibular Disorders of the Bárány Society. J. Vestib. Res..

[2] World Health Organization (2022). ICD-11: International Classification of Diseases.

[3] Popkirov S., Staab J.P., Stone J. (2018). Persistent postural-perceptual dizziness (PPPD): A common, characteristic and treatable cause of chronic dizziness. Pract. Neurol..

[4] Staab J.P. (2020). Persistent Postural-Perceptual Dizziness. Semin. Neurol..

[5] Dieterich M., Staab J.P., Brandt T. (2016). Functional (psychogenic) dizziness. Handb. Clin. Neurol..

[6] Steensnaes M.H., Knapstad M.K., Goplen F.K., Berge J.E. (2023). Persistent Postural-Perceptual Dizziness (PPPD) and quality of life: A cross-sectional study. Eur. Arch. Otorhinolaryngol..

[7] Habs M., Strobl R., Grill E., Dieterich M., Becker-Bense S. (2020). Primary or secondary chronic functional dizziness: Does it make a difference? A DizzyReg study in 356 patients. J. Neurol..

[8] Huppert D.K., Brandt T. (1995). Phobic postural vertigo (154 patients): Its association with vestibular disorders. J. Audiol. Med..

[9] Waterston J., Chen L., Mahony K., Gencarelli J., Sturat G. (2021). Persistent Postural-Perceptual Dizziness: Precipitating Conditions, Comorbidities and Treatment with Cognitive Behavioral Therapy. Front. Neurol..

[10] Gambacorta V., D’Orazio A., Pugliese V., Di Giovanni A., Ricci G., Faralli M. (2022). Persistent Postural Perceptual Dizziness in Episodic Vestibular Disorders. Audiol. Res..

[11] Strupp M., Bisdorff A., Furman J., Hornibrook J., Jahn K., Maire R., Newman-Toker D., Magnusson M. (2022). Acute unilateral vestibulopathy/vestibular neuritis: Diagnostic criteria. J. Vestib. Res..

[12] Kammerlind A.S., Ledin T.E., Skargren E.I., Odkvist L.M. (2005). Long-term follow-up after acute unilateral vestibular loss and comparison between subjects with and without remaining symptoms. Acta Otolaryngol..

[13] Cousins S., Cutfield N.J., Kaski D., Palla A., Seemungal B.M., Golding J.F., Staab J.P., Bronstein A.M. (2014). Visual dependency and dizziness after vestibular neuritis. PLoS ONE.

[14] Nuti D., Zee D.S., Mandalà M. (2020). Benign paroxysmal positional vertigo: What we do and do not know. Semin. Neurol..

[15] Von Brevern M., Radtke A., Lezius F., Feldmann M., Ziese T., Lempert T., Neuhauser H. (2007). Epidemiology of benign paroxysmal positional vertigo: A population-based study. J. Neurol. Neurosurg. Psychiatry.

[16] Bhattacharyya N., Gubbels S.P., Schwartz S.R., Edlow J.A., El-Kashlan H., Fife T., Holmberg J.M., Mahoney K., Hollingsworth D.B., Roberts R. (2017). Clinical Practice Guideline: Benign Paroxysmal Positional Vertigo (Update). Otolaryngol. Head Neck Surg..

[17] Kim J.S., Zee D.S. (2014). Clinical practice. Benign paroxysmal positional vertigo. N. Engl. J. Med..

[18] Von Brevern M., Bertholon P., Brandt T., Fife T., Imai T., Nuti D., Newman-Toker D. (2015). Benign paroxysmal positional vertigo: Diagnostic criteria. J. Vestib. Res..

[19] Headache Classification Committee of the International Headache Society (2018). The International Classification of Headache Disorders. Cephalalgia.

[20] Lempert T., Olesen J., Furman J., Waterston J., Seemungal B., Carey J., Bisdorff A., Versino M., Evers S., Newman-Toker D. (2012). Vestibular migraine: Diagnostic criteria. J. Vestib. Res..

[21] Yao K., Zu H.B. (2023). Isolated transient vertigo due to TIA: Challenge for diagnosis and therapy. J. Neurol..

[22] Trinidade A., Cabreira V., Goebel J.A., Staab J.P., Kaski D., Stone J. (2023). Predictors of persistant-perceptual dizziness (PPPD) and similar forms of chronic dizziness precipitated by peripheral vestibular disorders: A systematic review. J. Neurol. Neurosurg. Psychiatry.

[23] Toshishige Y., Kondo M., Kabaya K., Watanabe W., Fukui A., Kuwabara J., Nakayama M., Iwasaki S., Furukawa T.A., Akechi T. (2020). Cognitive-behavioural therapy for chronic subjective dizziness: Predictors of improvement in Dizziness Handicap Inventory at 6 months posttreatment. Acta Oto-Laryngol..

[24] Brandt T., Huppert D., Strupp M., Dieterich M. (2015). Functional dizziness: Diagnostic keys and differential diagnosis. J. Neurol..

[25] Kabaya K., Tamai H., Okajima A., Minakata T., Kondo M., Nakayama M., Iwasaki S. (2022). Presence of exacerbating factors of persistent perceptual-postural dizziness in patients with vestibular symptoms at initial presentation. Laryng. Investig. Otolaryngol..

[26] Murofushi T., Nishimura K., Tsubota M. (2022). Isolated Otolith Dysfunction in Persistent Postural-Perceptual Dizziness. Front. Neurol..

[27] Molnár A., Maihoub S., Mavrogeni P., Tamás L., Szirmai Á. (2022). Depression scores and quality of life of vertiginous patients, suffering from different vestibular disorders. Eur. Arch. Otorhinolaryngol..

[28] Best C., Eckhardt-Henn A., Tschan R., Dieterich M. (2009). Psychiatric morbidity and comorbidity in different vestibular vertigo syndromes. Results of a prospective longitudinal study over one year. J. Neurol..

[29] Tropiano P., Lacerenza L.M., Agostini G., Barboni A., Faralli M. (2021). Persistent postural perceptual dizziness following paroxysmal positional vertigo in migraine. Acta Otorhinolaryngol. Ital..

[30] Neuhauser H.K. (2016). The epidemiology of dizziness and vertigo. Handb. Clin. Neurol..

[31] Powell G., Derry-Sumner H., Rajenderkumar D., Rushton S.K., Sumner P. (2020). Persistent postural perceptual dizziness is on a spectrum in the general population. Neurology.

[32] Teggi R., Quaglieri S., Gatti O., Benazzo M., Bussi M. (2013). Residual dizziness after successful repositioning maneuvers for idiopathic benign paroxysmal positional vertigo. ORL.

[33] Martellucci S., Pagliuca G., De Vincentiis M., Greco A., De Virgilio A., Nobili Benedetti F.M., Gallo A. (2016). Features of residual dizziness after canalith repositioning procedures for benign paroxysmal positional vertigo. Otolaryngol. Head Neck Surg..

[34] Seok J., Lee H.M., Yoo J.H., Lee D.K. (2008). Residual dizziness after successful repositioning treatment in patients with benign paroxysmal positional vertigo. J. Clin. Neurol..

[35] Faralli M., Lapenna R., Giommetti G., Pellegrino C., Ricci G. (2016). Residual dizziness after the first BPPV episode: Role of otolithic function and of a delayed diagnosis. Eur. Arch. Oto-Rhino-Laryngol..

[36] Parham K., Kuchel G.A. (2016). A geriatric perspective on benign paroxysmal positional vertigo. J. Am. Geriatr. Soc..

[37] Balatsouras D.G., Koukoutsis G., Fassolis A., Moukos A., Apris A. (2018). Benign paroxysmal positional vertigo in the elderly: Current insights. Clin. Intervent. Aging.

[38] Piker E.G., Jacobson G.P. (2014). Self-report symptoms differ between younger and older dizzy patients. Otol. Neurotol..

[39] Nahm H., Han K., Shin J.E., Kim C.H. (2019). Benign Paroxysmal Positional Vertigo in the Elderly: A Single-center Experience. Otol. Neurotol..

[40] Jumani K., Powell J. (2017). Benign paroxysmal positional vertigo: Management and its impact on falls. Ann. Otol. Rhinol. Laryngol..

[41] Casani A.P., Gufoni M., Capobianco S. (2021). Current Insights into Treating Vertigo in Older Adults. Drugs Aging.

[42] Gámiz M.J., Lopez-Escamez J.A. (2004). Health-related quality of life in patients over sixty years old with benign paroxysmal positional vertigo. Gerontology.

[43] Lopez-Escamez J.A., Gamiz M.J., Fernandez-Perez A., Gomez-Fiñana M., Sanchez-Canet I. (2003). Impact of treatment on health-related quality of life in patients with posterior canal benign paroxysmal positional vertigo. Otol. Neurotol..

[44] Bronstein A.M., Golding J.F., Gresty M.A., Mandalà M., Nuti D., Shetye A., Silove Y. (2010). The social impact of dizziness in London and Siena. J. Neurol..

[45] Bruss D., Abouzari M., Sarna B., Goshtasbi K., Lee A., Birkenbeuel J., Djalilian H.R. (2021). Migraine Features in Patients with Recurrent Benign Paroxysmal Positional Vertigo. Otol. Neurotol..

